# The glutamic residue at position 402 in the C-terminus of Newcastle disease virus nucleoprotein is critical for the virus

**DOI:** 10.1038/s41598-017-17803-2

**Published:** 2017-12-12

**Authors:** Xiaohui Yu, Jinlong Cheng, Zirong He, Chuang Li, Yang Song, Jia Xue, Huiming Yang, Rui Zhang, Guozhong Zhang

**Affiliations:** 1 Key Laboratory of Animal Epidemiology of the Ministry of Agriculture, College of Veterinary Medicine, China Agricultural University, Beijing, 100193 China; 2 Department of Basic Veterinary Medicine, College of Veterinary Medicine, China Agricultural University, Beijing, 100193 China

## Abstract

The nucleocapsid proteins (NPs) of Newcastle disease virus (NDV) and other paramyxoviruses play an important functional role during genomic RNA replication. Our previous study showed that the NP-encoding gene significantly influenced viral replication. Here, we investigated the roles of certain amino acid residues in the NP C-terminus in viral replication and virulence. Results showed that the glutamic acid residue at position 402 (E402) in the C-terminus of the NP is critical for RNA synthesis in the NDV mini-genome system. Mutation of E402 resulted in larger viral plaques that appeared more quickly, and increased the virulence of NDV. Further study indicated that the mutant virus had increased RNA levels during the early stages of virus infection, but that RNA replication was inhibited at later time points. These findings increase our knowledge of viral replication and contribute to a more comprehensive understanding of the virulence factors associated with NDV.

## Introduction

Newcastle disease (ND), caused by Newcastle disease virus (NDV), is a severe infectious disease that affects over 250 bird species^1^. NDV is a member of the genus *Avulavirus*, within the family Paramyxoviridae^2^. The NDV genome encodes six structural proteins: nucleoprotein (NP), phosphoprotein (P), matrix protein (M), fusion protein (F), hemagglutinin- neuraminidase (HN), and large polymerase protein (L)^3^. The M, F, and HN proteins are associated with the viral envelope. The M protein is involved in morphogenesis and budding. The F and HN proteins mediate entry and release^4,5^. The viral RNA genome is always found tightly associated with NP, and the NP-RNA complex associates with the viral RNA-dependent RNA polymerase (RdRp) complex, which consists of P and L proteins, to form an active ribonucleoprotein (RNP) complex that is necessary for transcription and replication^6–10^.

NP is the most abundant protein in virus particles, and plays an important role in viral transcription and replication. It consists of two major domains: a highly conserved structured N-terminal region (N_CORE_), which forms the globular body, and a C-terminal region (N_TAIL_), which extends from the N-terminal body^11–13^. The N_CORE_ contains all of the components necessary for NP self-assembly and RNA-binding to form the NP-RNA complex, while the C-terminal tail is mainly responsible for the interaction of the NP-RNA complex with the P protein^10,11,14–16^. Thus, binding of the NP-RNA template to the RdRp complex for viral RNA synthesis mainly occurs via the C-terminus of NP^17,18^.

Several studies have investigated the specific domains responsible for the different functions of NP in non-segmented negative-stranded RNA viruses, but there are discrepancies between the different studies. Several studies indicated that the N-terminus, but not the C-terminus, of NP is required for NP-RNA complex formation and self-assembly, while another study showed that the C-terminus of NP is an important determinant of encapsidation and RNA synthesis^19–22^. Both the N_CORE_ and N_TAIL_ regions of the NP are likely to play key roles in binding to P protein^18,23,24^. However, there are very few studies on this aspect of NP that are specific to NDV, and the functional domains of NP require further exploration. Furthermore, our previous study showed that exchanging the NP gene between NDV strains LaSota (genotype II) and SG10 (genotype VII) had a significant effect on the replication kinetics of the resulting chimeric viruses^25^.

Here, we investigated the role of the NDV NP C-terminus in viral replication by generating a series of truncated and alanine-scanning mutants, and testing the associated activity of the mutant proteins. Our results suggested that the glutamic acid residue at position 402 (E402) in the C-terminus is critical for RNA synthesis. Mutation of this critical residue led to increased levels of mRNA transcription during the early stages of virus infection, but inhibited RNA replication at later time points. Experiments using the mutant strains also revealed that the E402 residue negatively regulates the virulence of NDV. Therefore, this study identifies a specific site that plays a key role in maintaining NDV NP function.

## Results

### Prediction of the functional sites in the NDV NP

**Figure 1:**
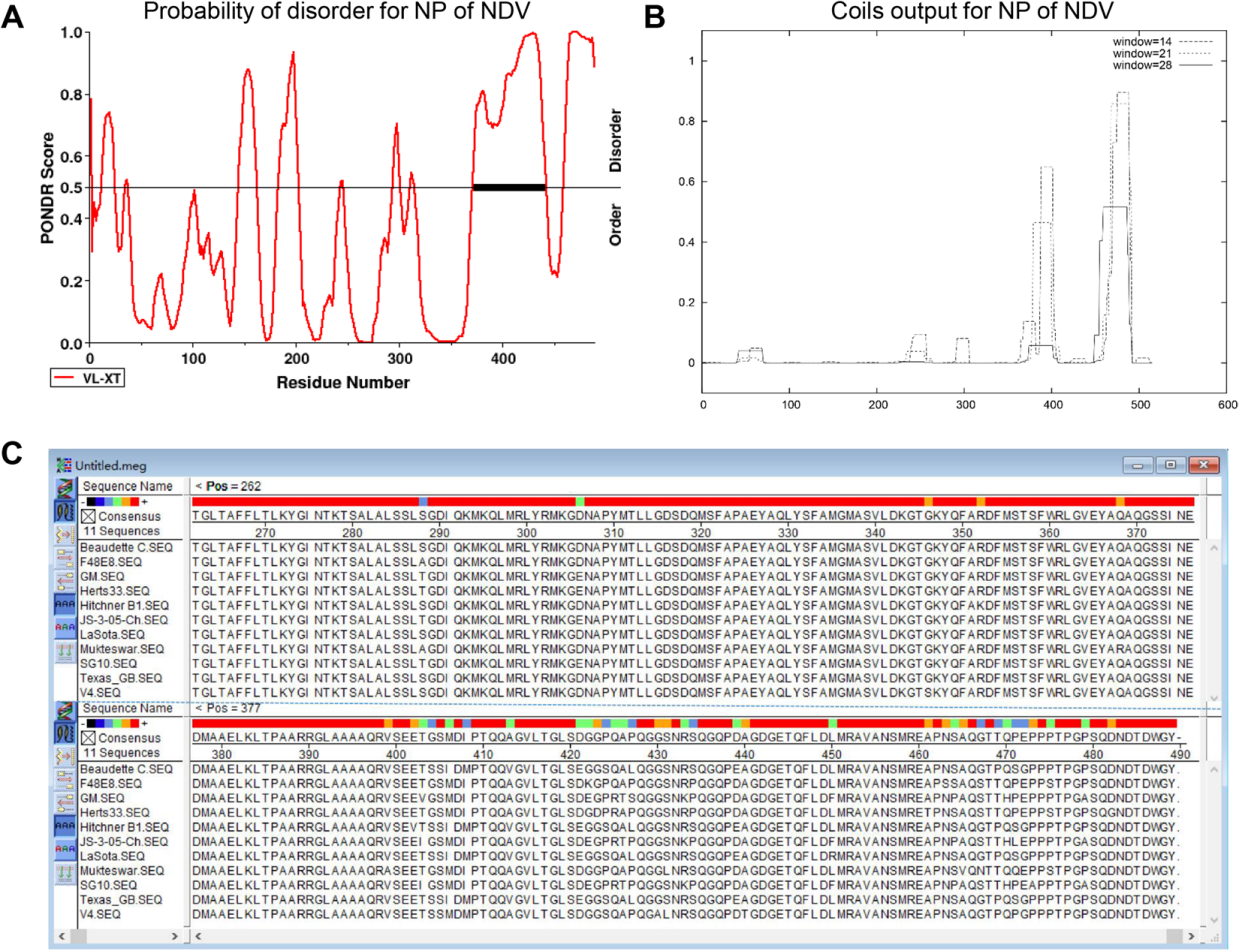
Prediction of the functional sites in the Newcastle disease virus (NDV) nucleoprotein (NP). (**A**) The PONDR program was used to determine the potential structural disorder within NDV NP. The disorder prediction value for a given residue is plotted against the residue number. The significance threshold, above which residues are considered to be disordered, was set to 0.5. The bold black line indicates the main structurally disordered region. (**B**) COILS prediction of the potential coiled-coil motif of NDV NP. The graph displays the probability of coiled-coil formation as a function of residue number using 14-amino-acid, 21-amino-acid, and 28-amino-acid scan windows. (**C**) The MegAlign program was used to analyze the amino acid sequence differences among genotypes.

A bioinformatics analysis of the potential function sites in the NDV NP was performed using three separate online programs. Previous studies indicated that interactions between the NP and P proteins are regulated by an IDR^26^. The PONDR prediction program was used to predict the structural disorder of the NP, and revealed two IDRs in the C-terminal region. One was located at the C-terminus (amino acids 460–489), while the other was located in a particularly highly disordered region (amino acids 360–430) of the NP C-terminus (Fig. 1A). A coiled-coil motif within a protein usually has a special function, such as modulating the interactions of different proteins or acting as a molecular recognition system. Thus, we used the COILS program to determine if such a motif was present in the NDV NP sequence^27^. The COILS analysis revealed two high-probability coiled-coil motifs within the C-terminus of NP (approximate amino acid locations 380–410 and 470–489) (Fig. 1B). In addition, the complete amino acid sequences of the NP genes from NDV strains of different genotypes were analyzed using ClustalW, which produced a result similar to those obtained using the first two methods. The analysis showed that while the N-terminus of NP (approximate amino acids 1–400) is highly conserved, the C-terminus has obvious differences between genotypes (Fig. 1C). Thus, the C-terminal region is likely to be required for the function of the NP, and for regulating the interaction of NP with other proteins.

### The NP amino acids 402–408 hardly support NDV mini-genome expression

**Figure 2:**
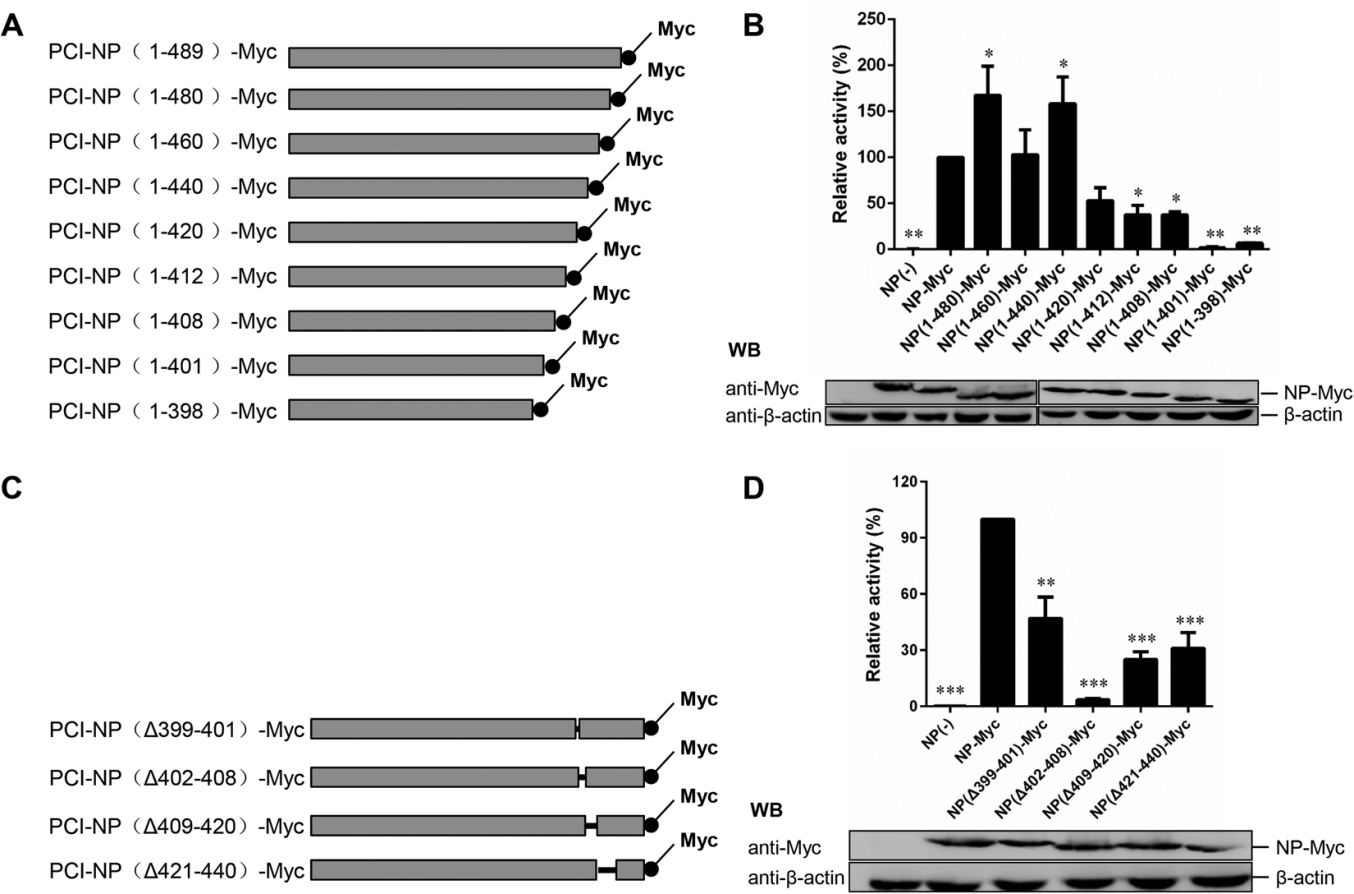
Newcastle disease virus (NDV) mini-genome expression of nucleoprotein (NP) mutants. The NDV mini-genome expression of full-length NDV NP was compared with NP truncation mutants (**A**,**B**) and NP deletion mutants (**C**,**D**). (**A** and **C**) Schematic representation of full-length NDV NP-Myc and NP-Myc mutants. (**B** and **D**) Mini-genome RNA synthesis activity of the NP mutants. BSR T7 cells were transfected with plasmids as described in the Materials and Methods. The relative luciferase expression levels of cells transfected with plasmids encoding NP-Myc were defined as 100%. Expression of NP-Myc and NP-Myc mutants was detected via western blotting using an anti-Myc antibody. The gels are uncropped images. Asterisks indicate a statistically significant difference between the titers of the chimeric virus and the parental virus. *P*-values were calculated based on a two-tailed, unpaired *t*-test (95% confidence levels). *P = 0.01–0.05; **P = 0.001–0.01; ***P = 0.0001–0.001*. n* = *3*.

To examine the function of the NP C-terminus, a series of plasmids encoding progressively truncated C-terminal mutants of NP with a Myc tag were constructed based on the predicted results (Fig. 2A), and a NDV mini-genome system was produced^25^. We first generated a plasmid encoding wild-type NP with a Myc tag to confirm that the luciferase expression of the Myc-tagged NP was similar to that of untagged NP, suggesting that NP-Myc has a similar function to the untagged NP (data not shown). Next, we measured the RNA synthesis activities of the progressively truncated C-terminal NP mutants in the mini-genome system. As shown in Fig. 2B, mutants NP(1–480)-Myc, NP(1-460)-Myc, and NP(1-440)-Myc had luciferase expression that was higher than or similar to that of NP-Myc, while the luciferase expression of NP(1-420)-Myc, NP(1-412)-Myc, NP(1-408)-Myc, NP(1-401)-Myc, and NP(1-398)-Myc was lower than that of NP-Myc. The luciferase expression of NP(1-401)-Myc and NP(1-398)-Myc was particularly low, with levels that were almost undetectable.

To further confirm the main functional sites within amino acid region 399–440, we evaluated the RNA synthesis activity of NP(Δ421-440)-Myc, NP(Δ409-420)-Myc, NP(Δ402-408)-Myc and NP(Δ399-401)-Myc (Fig. 2C). Similar to the above results, all of these mutants caused demonstrated reduced luciferase expression compared with NP-Myc in the mini-genome system, with the lowest levels of luciferase recorded for the NP(Δ402-408)-Myc mutant (Fig. 2D). This suggested that amino acids 402–408 play an important role in the function of the NDV NP protein. The different Myc-tagged NP protein mutants were also analyzed by western blotting using a monoclonal anti-Myc antibody, which confirmed that the correct mutant NP proteins were present, and that the expression levels were comparable (Fig. 2B and D, bottom panel).

### Effects of mutating E402 to alanine in the NDV mini-genome system

**Figure 3:**
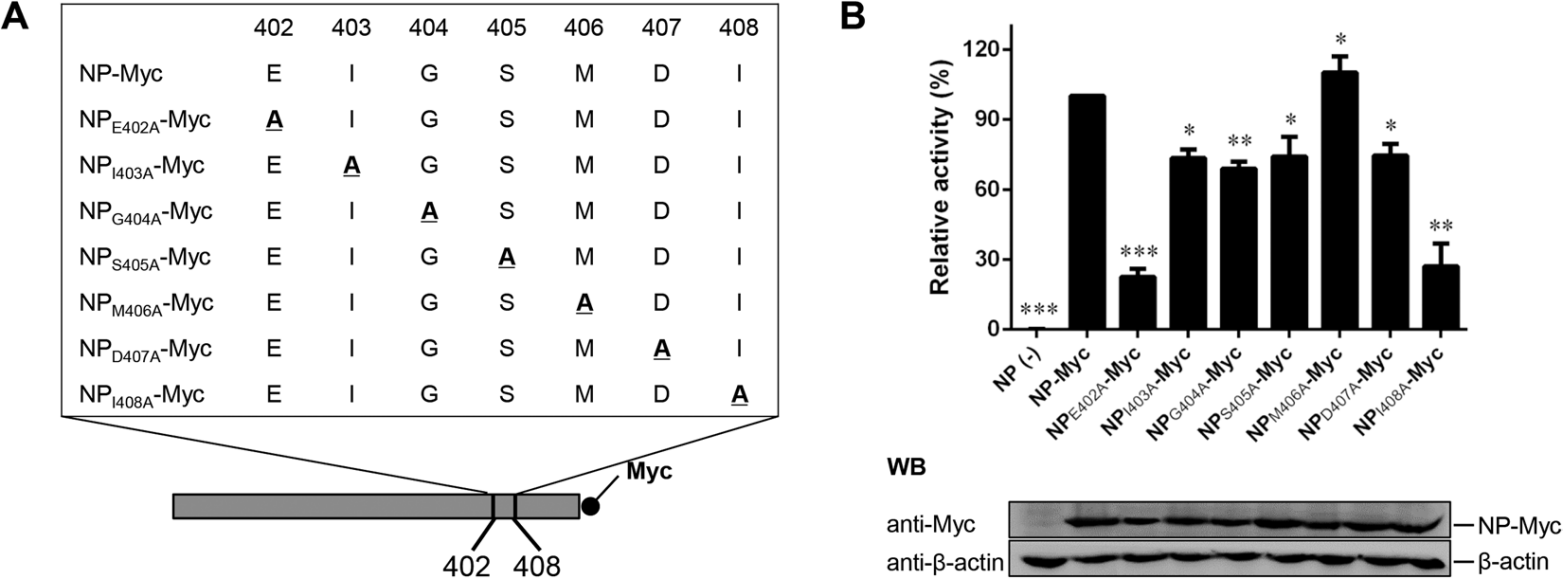
Effects of mutating E402 to alanine in the Newcastle disease virus mini-genome system. (**A**) Myc-tagged alanine-scanning nucleoprotein (NP) mutants were used. Serial residues within NP amino acid region 402–408 were replaced with alanine (shown in bold and underlined). The names of the mutants are shown on the left. (**B**) Mini-genome RNA synthesis activities of the NP mutants. BSR T7 cells were transfected with plasmids as described in the Materials and Methods. Relative luciferase expression levels were measured, and the expression of NP-Myc and the NP-Myc mutants was detected via western blotting. The gels are uncropped images. Asterisks indicate a statistically significant difference between the viral titers of the chimeric virus and the parental virus. *P*-values were calculated based on a two-tailed, unpaired *t*-test (95% confidence levels). *P = 0.01–0.05; **P = 0.001–0.01; ***P = 0.0001–0.001*. n* = *3*.

To identify the precise amino acid residue that is indispensable for the RNA synthesis function of NP, we generated a further seven plasmids encoding Myc-tagged NP mutants with unique alanine (A) substitutions in amino acid region 402–408 (Fig. 3A). We then evaluated the resulting luciferase expression in BSR T7 cells expressing P and L proteins, mini-genome, and NP-Myc, or one of the individual alanine-scanning NP mutants. Mutants NP_I403A_-Myc, NP_G404A_-Myc, NP_S405A_-Myc, NP_M406A_-Myc, and NP_D407A_-Myc had luciferase expression levels similar (75–110%) to those of NP-Myc, while the luciferase expression of mutant NP_I408A_-Myc was only 25% of that of NP-Myc. However, the relative luciferase expression of NP_E402A_-Myc was only about 8% of that of NP-Myc (Fig. 3B, upper panel), although the expression levels of all the mutant NP proteins were similar (Fig. 3B, bottom panel). These findings indicate that NP_E402A_ showed very little activity in the mini-genome assay.

### Recovery of recombinant chimeric virus with a NP E402A mutation

To further determine the role of amino acid residue E402 in the virus, we constructed a recombinant NDV strain harboring an E402A mutation based on the cDNA of NDV strain SG10 (rNDV-WT). The recombinant virus, named rNDV-NP_E402A_, was viable, and could be rescued by taking advantage of reverse genetics. To ensure the genetic stability of the rescued virus, it was serially passaged 10 times in 9-day-old specific-pathogen-free (SPF) chicken eggs. The mutant virus was amplified by reverse-transcription PCR (RT-PCR) and then sequenced, with the results confirming the presence of the introduced mutations and the absence of adventitious mutations (data not shown).

### Biological characteristics of the recombinant virus

**Figure 4:**
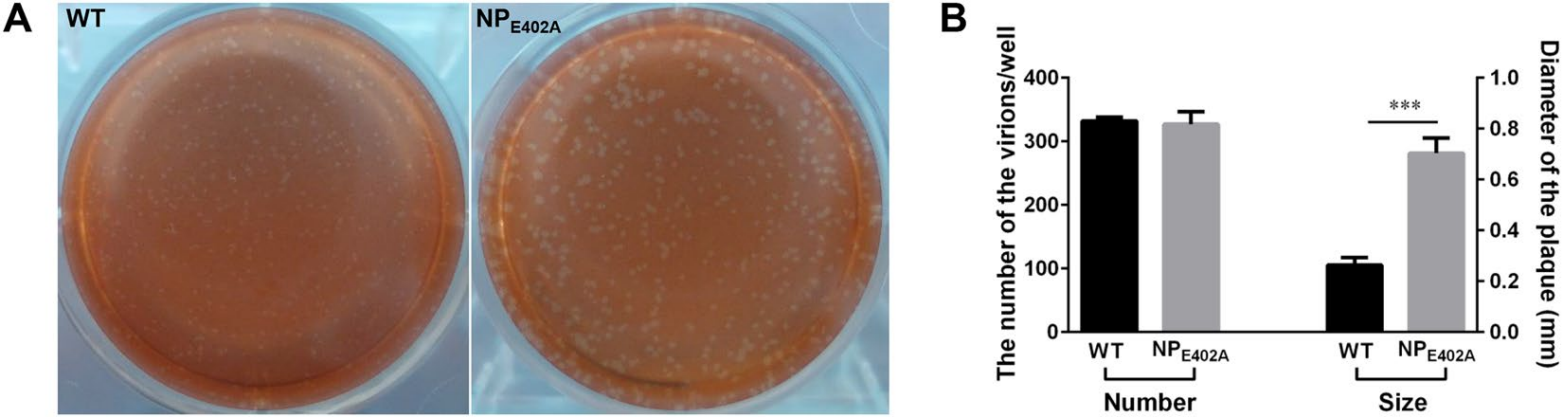
Plaque morphologies of the wild-type and mutant viruses in BSR T7 cells. (**A**) The shape and size of plaques formed by these viruses. (**B**) Summary of the average plaque number and size for each virus. *P*-values were calculated based on a two-tailed, unpaired *t*-test (95% confidence levels). *P = 0.01–0.05; **P = 0.001–0.01; ***P = 0.0001–0.001*. n* = *3*.

The biological characteristics of the mutant and wild-type NDVs were then compared (Table 1). The results revealed that the rNDV-NP_E402A_ mutant had similar viral titers to those of the wild-type (rNDV-WT) in both embryos and cells (Log_10_ 9.37 and 8.64 EID_50_/ml; Log_10_ 8.57 and 9.0 TCID_50_/ml, respectively). In addition, the pathogenicity of the two viruses in chicken embryos and 1-day-old chickens was comparable. We further evaluated the cytopathic effects by measuring the numbers and sizes of the plaques formed by the chimeric mutated virus rNDV-NP_E402A_ and rNDV-WT on BSR T7 cell monolayers. In plaque assays, there was a more rapid and extensive cytopathic effect in cells infected with rNDV-NP_E402A_ (average plaque diameter: 0.70 ± 0.06 mm) compared with the cells infected with rNDV-WT (average plaque diameter: 0.26 ± 0.03 mm), although the numbers of the plaques were similar for the two strains (Fig. 4A and B).

**Table 1:** Biological characteristics of the wild-type and mutant viruses.

Virus	Pathogenicity		Virus titer	
	ICPI score^a^	MDT (h)^b^	Log_10_EID_50_/ml	Log_10_TCID_50_/ml
WT	1.86	42	8.64	9.00
NPE402A	1.81	36	9.37	8.57

^a^Intracerebral pathogenicity index (ICPI): virulent strains, 1.50–2.00; moderately virulent strains, 0.70–1.50; avirulent strains, 0.00–0.70.

^b^Mean death time (MDT): virulent strains, < 60 h; moderately virulent strains, 60–90 h; avirulent strains, >90 h.

### Replication and pathogenicity in 4-week-old chickens

**Figure 5:**
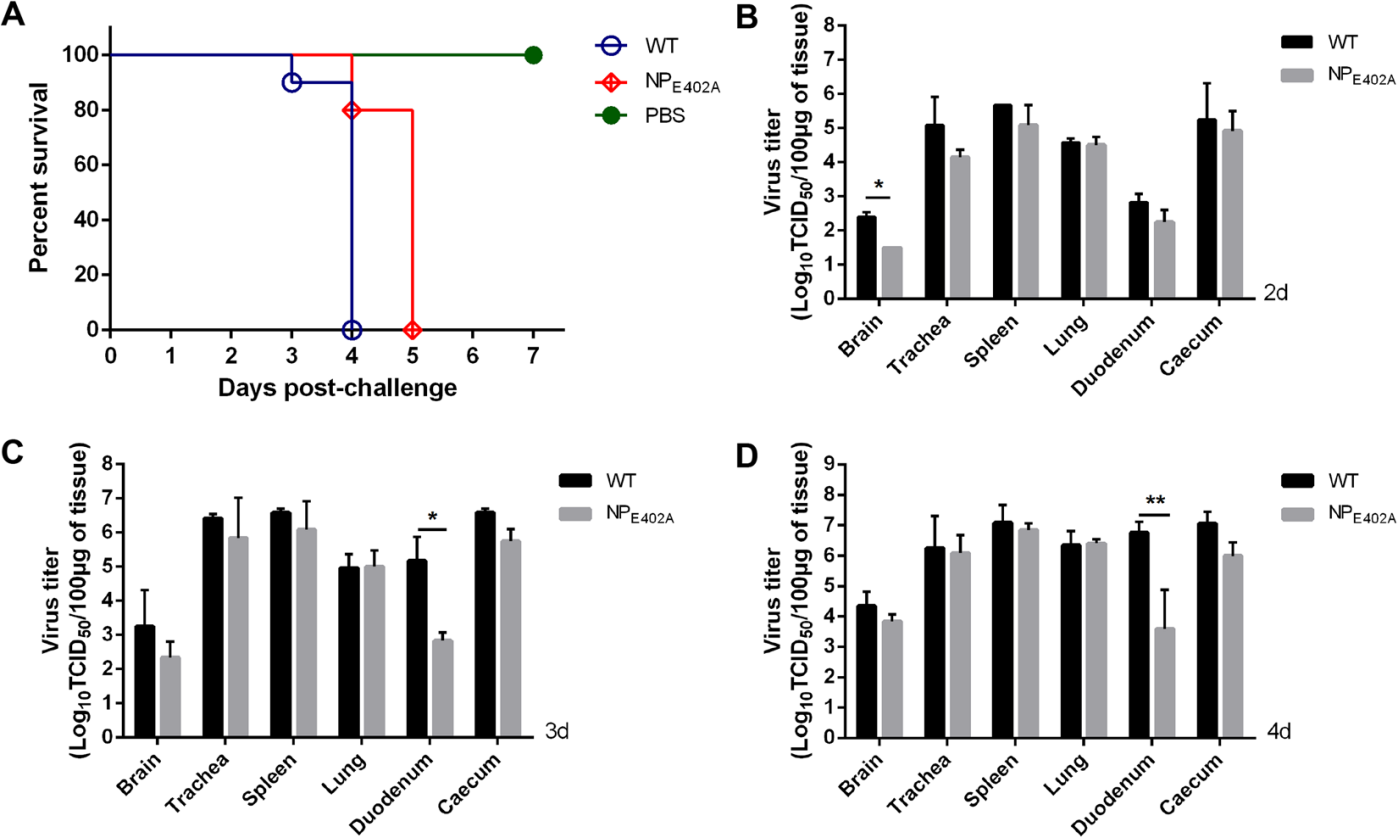
Survival curves and replication of rNDV-NP_E402A_ and rNDV-WT in 4-week-old chickens. (**A**) Survival curves of 4-week-old specific pathogen-free chickens (n = 10). Birds were infected with rNDV-WT or rNDV-NP_E402A_. (**B**–**D**) Replication of the two viruses in 4-week-old chickens. Birds were inoculated with 10^5^EID_50_/100 *μ*l of rNDV-NP_E402A_ or rNDV-WT through the eye drop/intranasal (ED/IN) route, and sacrificed at 2 (**B**), 3 (**C**), or 4 (**D**) days post-inoculation (n = 2). The indicated tissues were collected, and viral titers were determined in DF-1 cells. Asterisks indicate a statistically significant difference between the titers of the chimeric virus and the parental virus. *P*-values were calculated based on a two-tailed, unpaired *t*-test (95% confidence levels). *P = 0.01–0.05; **P = 0.001–0.01.

The replication and pathogenicity of rNDV-WT and rNDV-NP_E402A_ was evaluated in 4-week-old SPF white Leghorn chickens by inoculating each bird with 10^5^ EID_50_/100 *μ*l of viral particles via the eye drop/intranasal (ED/IN) route. The resulting survival curves are shown in Fig. 5A. Birds inoculated with rNDV-WT exhibited a slight depression at 2 dpi, severe depression (2/10), wing drop/incoordination (4/10), prostration (3/10), and death (1/10) at 3 dpi, and 100% mortality by 4 dpi. At necropsy, all euthanized chickens showed severe hemorrhage in the throat and trachea, duodenum mucosa, and proventriculus, necrosis in the caecum, and white, necrotic, pinpoint foci in the spleen at 3–4 dpi. In comparison, the birds inoculated with rNDV-NP_E402A_ developed moderate illness by 3 dpi, severe illness by 4 dpi, and 100% mortality by 5 dpi (Fig. 5A). Virus titration assays (Fig. 5B, C, and D) showed that both rNDV-WT and rNDV-NP_E402A_ could replicate in all organs, and that viral titers in the sampled tissues gradually increased over time. In general, the titers in most of the sampled tissues were similar between the two treatment groups, except for in the brain at 2 dpi (Fig. 5B) and in the duodenum at 3 (Fig. 5C) and 4 dpi (Fig. 5D), where the replication was significantly different (*P* < 0.05). These results indicated that rNDV-NP_E402A_ had a similar tissue tropism to the wild-type virus, but had a slightly different replication level in certain tissues.

**Figure 6:**
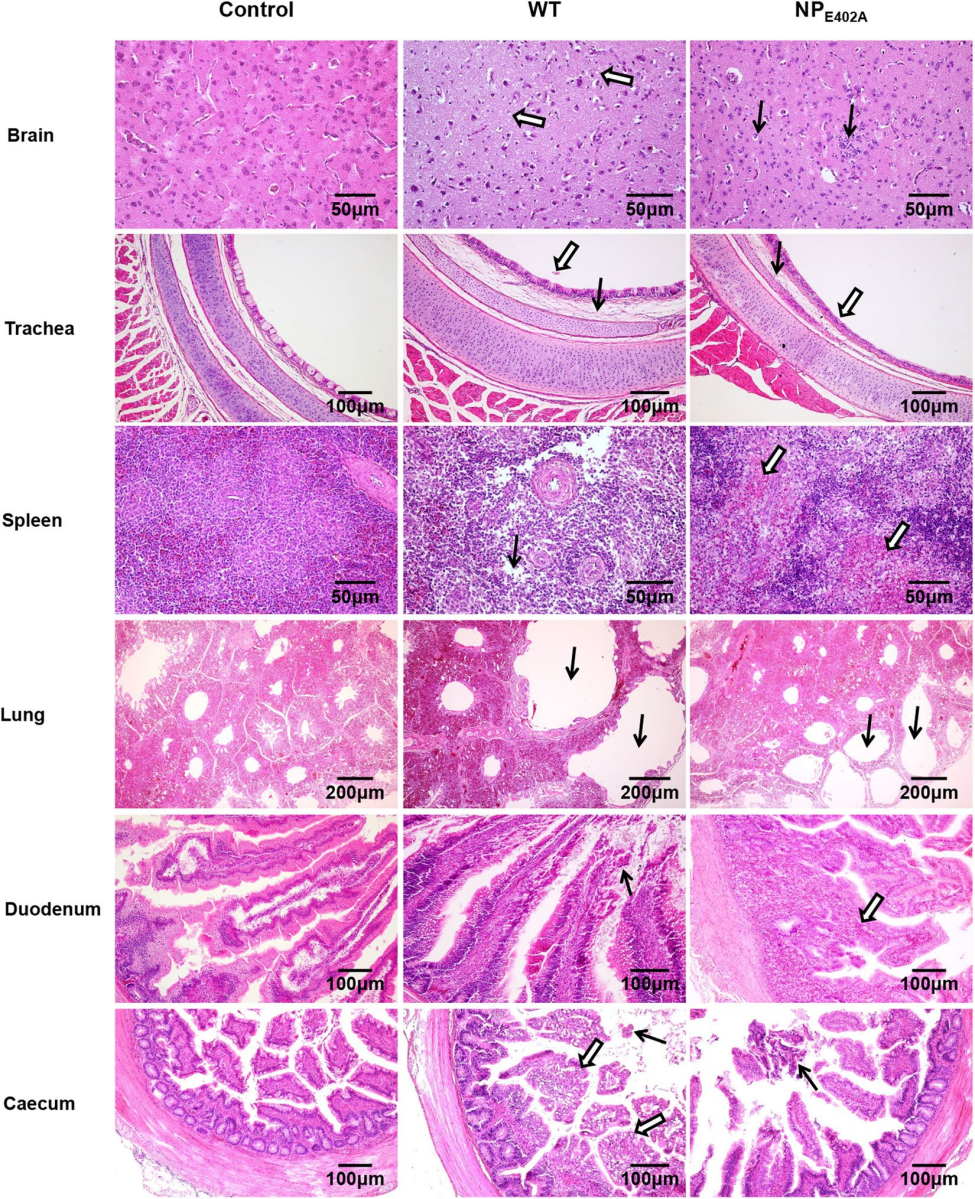
Histopathology of tissue samples collected from Newcastle disease virus (NDV)-inoculated 4-week-old chickens. Chickens were infected oculonasally with the wild-type virus (rNDV-WT) or the mutant (rNDV-NP_E402A_). Birds were sacrificed at 3 days post-inoculation, and the tissues were fixed with formalin, sectioned, and stained with hematoxylin and eosin.

Based on the histopathological analysis, both rNDV-WT and rNDV-NP_E402A_ caused the following moderate to severe histopathological changes in all of the sampled tissues (Fig. 6): loose brain tissue structure (empty arrows) with increased accumulation of microglial cells in the brain (black arrows); interstitial broadening and edema (black arrows) and slight mucosal epithelium shedding of the tracheal mucosa (empty arrows); lymphocyte necrosis (black arrows), multifocal confluent coagulative necrosis, and an increased inflammatory cell infiltration (empty arrows) in the spleen; extreme expansion in the bronchi of the lung (black arrows); mucosal epithelium shedding (black arrows) and inflammatory cell infiltration of the mucosal lamina propria in the duodenum (empty arrows); and mucosal epithelium shedding (black arrows) and increased goblet cells in the mucosal epithelium of the caecum. Overall, the two viruses caused similar histopathological changes in most tissues, except for in the spleen and brain. In the spleen, the tissue from the rNDV-NP_E402A_-inoculated group had particularly severe histopathological changes compared with that of the rNDV-WT-inoculated group (Fig. 6). As expected, no apparent histopathological changes were observed in any of the tissues from the control group, which was treated with 0.9% NaCl.

### Pathogenicity of wild-type and mutant NDV viruses in 1-day-old chicks

To compare the pathogenicity of the two viruses in 1-day-old chicks, an assay similar to the ICPI test was conducted. Birds were inoculated via the intracerebral route with different titers of rNDV-WT or rNDV-NPE402A (10^1^ EID_50_/50 *μ*l, 10^2^ EID_50_/50 *μ*l, or 10^3^ EID_50_/50 *μ*l per bird). The resulting mean scores are shown in Table 2. There was no difference between the two viruses at dose 10^3^ EID_50_/50 *μ*l. However, obvious differences were observed in the pathogenicity of the two viruses at doses 10^2^ EID_50_/50 *μ*l and 10^1^ EID_50_/50 *μ*l. This finding reveals that the rNDV-NP_E402A_ mutant virus is more virulent than the wild-type in 1-day-old chickens at lower viral titers.

**Table 2:** Pathogenicity of the wild-type and mutant viruses in 1-day-old chicks^a^.

Virus titers	Virus	
	WT	NP_E402A_
10^3^ EID_50_	1.66	1.65
10^2^ EID_50_	1.52	1.66
10 EID_50_	0.45	1.56

^a^The maximum score for velogenic strains is 2.0.

### Western blotting and immunoprecipitation

**Figure 7:**
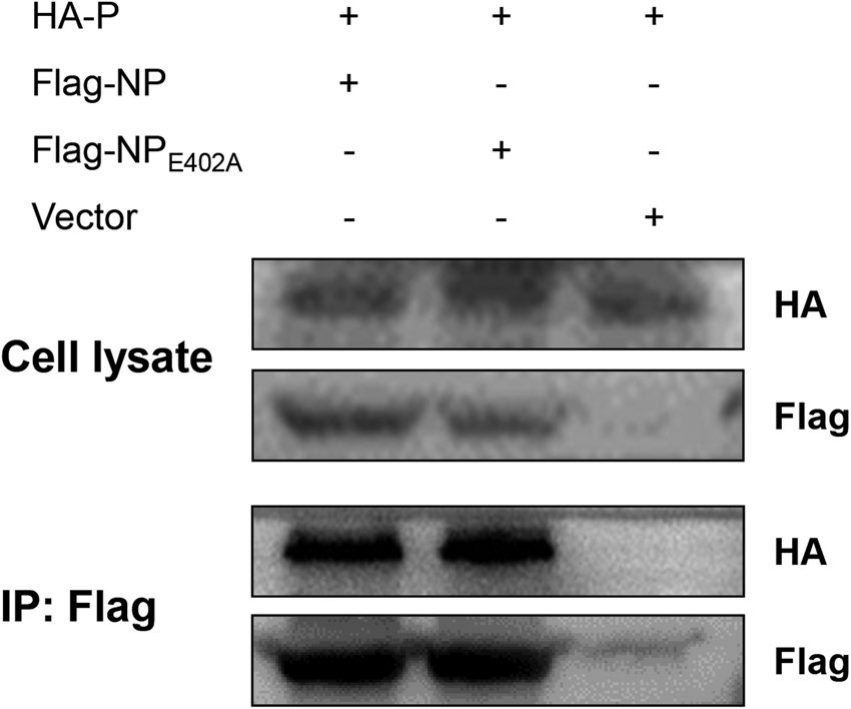
Nucleoprotein (NP) interacts with phosphoprotein (P) independent of the E402 site. 293 T cells were co-transfected with 2.5 *μ*g of plasmids expressing HA-tagged P protein, together with 2.5 *μ*g of plasmids expressing Flag-tagged NP or NP_E402A_. At 48 h post-transfection, cells were harvested and immunoprecipitated with anti-Flag M2 beads. The precipitates were separated by sodium dodecyl sulfate polyacrylamide gel electrophoresis, and P as well as NP proteins were detected in immunoblots using anti-HA or anti-Flag antibodies. The gels are uncropped images.

We next investigated whether the E402A substitution impacted on the interaction between the NP and P proteins, thereby affecting the pathogenicity of rNDV-NP_E402A_. We immunoprecipitated Flag-tagged NP and NP_E402A_ from 293 T cells overexpressing these proteins, and probed the precipitates from HA-tagged P protein (Fig. 7). The results showed that both NP and NP_E402A_ associated with P protein, and that the E402A substitution had no obvious effect on the interaction of NP with P protein. This revealed that the observed change in the pathogenicity of rNDV-NP_E402A_ was not related to the interaction between NP and P proteins.

### Viral replication in DF-1 cells

**Figure 8:**
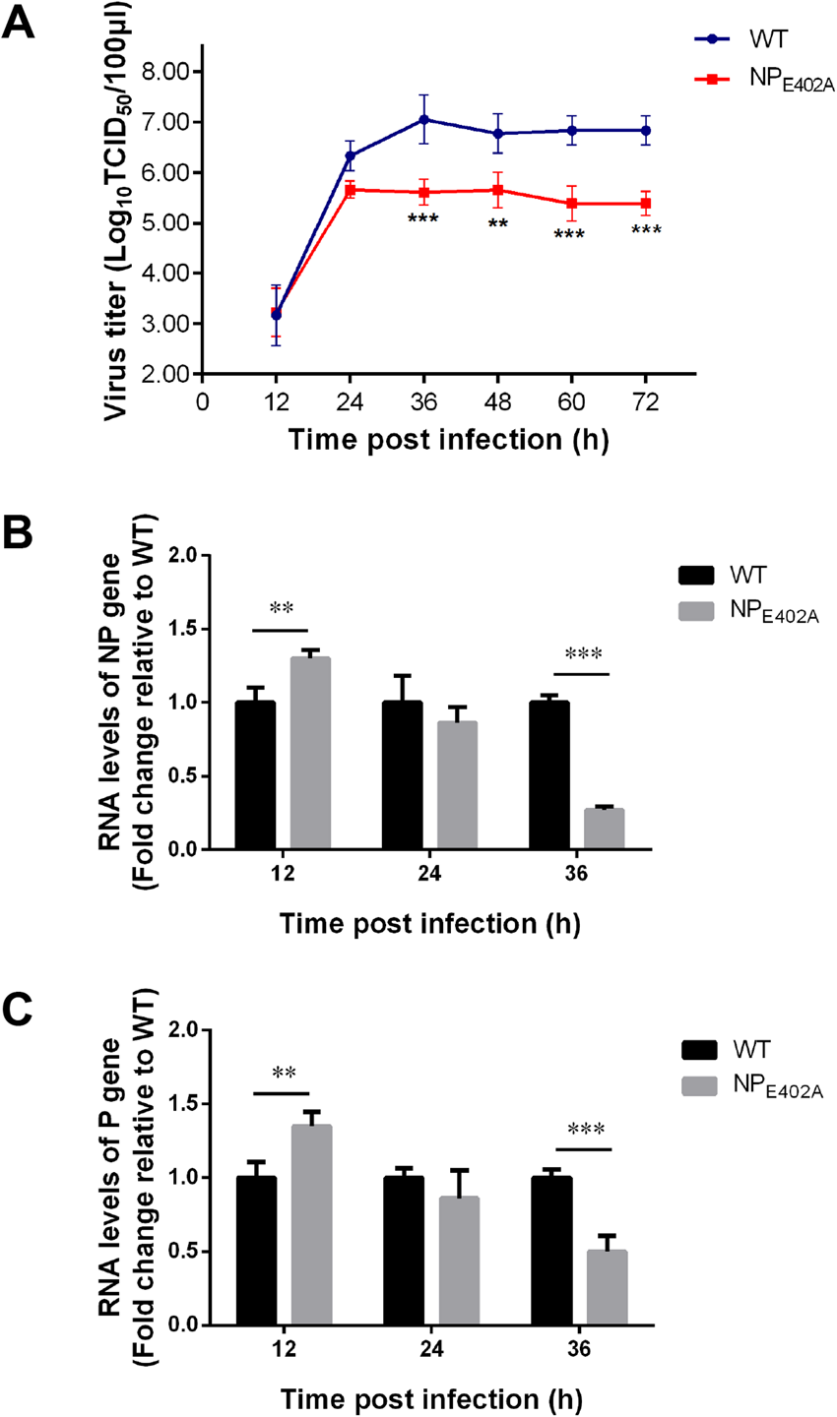
Replication levels and viral RNA synthesis of the recombinant viruses. (**A**) *In vitro* growth characterization of the wild-type and mutant viruses in DF-1 cells. DF-1 cells were infected with the indicated viruses at a multiplicity of infection (MOI) of 0.01, and the virus titers were determined at the indicated time points. Asterisks indicate a statistically significant difference in the titers of the chimeric virus and the parental virus. (**B** and **C**) Viral RNA synthesis in rNDV-NP_E402A_- and rNDV-WT-infected cells. DF-1 cells were infected with rNDV-WT or rNDV-NP_E402A_ at a MOI of 0.01, and total RNA was purified from infected cells at 12, 24, and 36 h post-infection. Levels of RNA corresponding to the nucleoprotein (**B**) and phosphoprotein (**C**) segments of Newcastle disease virus were measured in virus-infected cells by quantitative real-time PCR. RNA levels were normalized to those of GAPDH. *P*-values were calculated based on a two-tailed, unpaired *t*-test (95% confidence levels). *P = 0.01–0.05; **P = 0.001–0.01; ***P = 0.0001–0.001*. n* = *3*.

The growth kinetics of the two viruses were compared using multicycle growth curves in DF-1 cells (Fig. 8A). The rNDV-NP_E402A_ mutant showed a lower level of replication compared with the rNDV-WT. While rNDV-WT underwent exponential replication until 36 hpi, after which the replication level plateaued, exponential replication was only observed for the rNDV-NP_E402A_ mutant until 24 hpi. In addition, the rNDV-WT virus titer was significantly higher than that of rNDV-NP_E402A_ from 36 hpi to the end of the observation period (*P* < 0.01). Together, these results demonstrate that the E402A mutation in the C-terminus of the NP protein had a significant effect on the cytopathogenicity and replication capability of NDV.

### Effect of the NP E402 mutation on viral RNA synthesis

We next investigated RNA levels in the rNDV-WT and rNDV-NP_E402A_ strains using qRT-PCR. Individually, the levels of genomic RNA corresponding to the NP and P segments were similar across the two treatment groups for each of the time points (Fig. 8B and C). However, there were statistically significant differences in the relative RNA levels between cells infected with rNDV-WT and rNDV-NP_E402A_ at 12 hpi (*P* < 0.05), suggesting the faster replication by rNDV-NP_E402A_ during the early stages of infection. Despite this, the relative levels of viral RNA in the rNDV-NP_E402A_-infected samples were lower than those in rNDV-WT-infected cells at 24 hpi, and significantly lower at 36 hpi (*P* < 0.001). The data suggested that the E402A mutation specifically increased the levels of RNA expression in the earlier stages of infection, but reduced the RNA expression levels at later time points.

## Discussion

For NDV, vesicular stomatitis virus, and other non-segmented negative-stranded RNA viruses, the NP-RNA complex, consisting of the viral RNA genome and the NP protein, is necessary for transcription and replication^9^. Here, we examined the effects of substitutions in the amino acid residues located in the NDV NP C-terminal region on RNA synthesis regulation and viral virulence.

We first used three different online programs to identify possible functional sites. The intrinsically disordered region (IDR) has been shown to serve as an anchor point for binding with the polymerase complex in the NP C-terminus in several paramyxoviruses, including Nipah virus, Hendra virus, measles viruses, and human parainfluenza virus type 3 (HPIV3)^10,18,28^. However, little is known regarding this aspect of the NDV NP. Our analyses showed that the NP C-terminus contains two IDRs (amino acids 460–489 and amino acids 360–430) that were especially highly disordered. These regions were also identified by COILS program and MegAlign program analyses. We then explored the role of these IDRs in the RNA synthesis and virulence of NDV.

Using *in vivo* mini-genome RNA synthesis assays, we found that amino acids 399–440 in the NP C-terminus are important for RNA synthesis, especially amino acids 402–408. Interestingly, the NP mutant with a deletion of the 49 C-terminal amino acids had an ~160% increase in RNA synthesis activity compared with the wild-type NP. Similar results were obtained in other studies^10,29^. It is possible that the interaction between the NP-RNA template and the RdRp became relatively weak in these truncated proteins, allowing the polymerase greater access to the NP-RNA template during RNA synthesis. Thus, the full-length NP may result in a tight association between the P protein and the NP-RNA complex, which may not be beneficial for transcription and replication.

After identifying NP C-terminus amino acids 402–408 as being functionally important, we investigated which precise sites in this region are indispensable for RNA synthesis. Using individual alanine-scanning mutagenesis and the NDV mini-genome system, we found that the NP_E402A_-Myc mutant had the lowest levels of mini-replicon reporter gene expression, and that the reporter gene expression remained low despite gradual increases in the expression in related NP mutants. These findings confirm that amino acid E402 in the NP C-terminus is important for the RNA synthesis function of NP. These results are in agreement with previous studies on other non-segmented negative-stranded RNA viruses^9,20^.

To further investigate if the E402A mutation effects virus growth, a recombinant NDV harboring the E402A mutation was generated using reverse genetics. The two viruses showed comparable results in the EID_50_, TCID_50_, MDT, and ICPI assays (Table 1), but the rNDV-NP_E402A_ mutant developed faster and had 2.7-fold larger plaques compared with the rNDV-WT. Based on previous reports, we propose that NP_E402A_ from recombinant virus rNDV-NP_E402A_ had an increased ability to interact with the M protein^30–36^. This could influence the association between the M protein and the F and HN proteins, which are more efficient at inducing cell-cell fusion than the wild-type NP, resulting in the enlarged plaque. The increased plaque size may also be associated with differences in the innate immune response of the host cell to the different strains^37^.

We further determined the pathogenicity of the mutant virus rNDV-NP_E402A_ and its WT counterpart in 4-week-old SPF chickens inoculated via a natural route of infection. All infected chickens developed severe signs of illness, including severe lesions, and the mortality rate was 100% for both virus strains. A high viral titration was obtained from all harvested tissues, and except for the spleen, tissue samples from the rNDV-NP_E402A_-inoculated group showed severe histopathological changes that were comparable with those in the tissues from the rNDV-WT-inoculated group. As our earlier results suggested differences in the virulence between the two strains, we conducted intracerebral tests, which are similar to ICPI tests, to accurately compare the pathogenicity in 1-day-old chickens. The results showed that the rNDV-NP_E402A_ mutant was more virulent in 1-day-old chickens than the wild-type virus when lower virus titers were used for infection. We speculate that when hosts are infected with virulent viruses at a relatively high viral titer, the host dies too quickly to detect subtle differences in virulence.

Our analyses showed that the glutamic acid residue at position 402 (E402) in the NP C-terminus plays an important role in regulating viral virulence. To exploit the inherent mechanism responsible for the changes in virulence, we examined the interaction between NP and P proteins. The results showed that both NP and NP_E402A_ associated with P protein, and that the E402A mutation had no obvious effect on the interaction of NP with the P protein. This revealed that the observed change in the pathogenicity of rNDV-NP_E402A_ was not related to the interaction between NP and P proteins. We then investigated the replication ability of the two viruses in DF-1 cells using multicycle growth curves. Interestingly, the results showed significantly decreased replication by rNDV-NP_E402A_ compared with rNDV-WT from 36 hpi to the end of the observation period (*P* < 0.01 or 0.001).

Based on the differences in replication ability between the rNDV-NP_E402A_ and rNDV-WT strains, we next investigated the levels of viral RNA in infected DF-1 cells using qRT-PCR. The results showed that the E402A mutation specifically increased the levels of viral RNA expression at 12 hpi, but that expression was reduced at 36 hpi in the virus-infected cells. Previous studies also showed a differential effect on RNA synthetic processes^12,38^. The reason for the differential activity of replication and transcription of the same RNP template may be that two distinct complexes are involved, and that the different viral transcription and replication machinery recognize distinct regions of the RNP template^20,38–41^. The decreased replication of rNDV-NP_E402A_ compared with rNDV-WT may be the result of the enhanced cytopathic ability of strain rNDV-NP_E402A_. Because rNDV-NP_E402A_ developed faster and had a greater cytopathogenic effect, it caused serious damage to the host cells by 24 hpi, and the injured cells could not support effective viral RNA synthesis. Another reason may also be associated with the M protein. Some studies have shown that the M protein regulates viral RNA synthesis by interacting with the NP protein, which can stop viral RNA synthesis and promote viral particle production^35,42–44^. In rNDV-NP_E402A_, the close interaction between NP_E402A_ and M protein makes the incorporation of RNP complexes into progeny virions more efficiency. At the same time, more RNA synthesis is needed to allow transcription to occur, followed by replication. These findings also explain the increased levels of mRNA transcription in the early stages of virus infection.

In conclusions, we evaluated the roles of the specific amino acid residues in the C-terminus of the NDV NP in viral RNA synthesis and pathogenicity in the present study. Our findings reveal that the glutamic acid residue at position 402 (E402) in the NP C-terminus plays an important role in viral RNA synthesis. Mutation of this critical residue resulted in increased levels of RNA during the early stages of infection, but inhibited replication at later time points as a result of more severe cellular damage. The findings of the current study will be used to further investigate the distinction between transcription and replication, and will also contribute to a more comprehensive understanding of the virulence factors of NDV. However, it is not known if the NP E402 residue affects the encapsidation of RNA, the interaction of NP with the M protein, the formation of the NP-RNA template, or structural changes, and further studies are needed to elucidate this.

## Materials and Methods

### Cells and viruses

Chicken embryonic fibroblasts (DF-1) and 293 T cells were grown in Dulbecco’s modified Eagle’s medium (DMEM) (Gibco, Grand Island, NY, USA) supplemented with 10% (v/v) fetal bovine serum (FBS) (Gibco), and maintained in DMEM containing 2% FBS. Baby hamster kidney cells stably expressing T7 RNA polymerase (BSR T7 cells) were grown in DMEM containing 10% FBS and 1 mg/ml G418 (Invitrogen, Carlsbad, CA, USA). All cell lines were maintained at 37 °C in a 5% CO_2_ incubator (Thermo Forma, Marietta, OH, USA). NDV strain SG10 is a velogenic genotype VII virus, which was propagated in 9-day-old SPF embryonated chicken eggs via the allantoic cavity route^45^.

### Animals and ethics statement

SPF chicken eggs and chickens were purchased from Beijing Merial Vital Laboratory Animal Technology Co., Ltd. (Beijing, China). All chickens were housed in isolators at China Agricultural University (Beijing, China), and the rearing facilities were approved by the Beijing Administration Committee of Laboratory Animals under the auspices of the Beijing Association for Science and Technology (approval ID SYXK (Jing) 2013–0013). All of the animals used in this study were cared for in accordance with established guidelines, and the experimental protocols were specifically considered and approved by the Animal Welfare and Ethical Censor Committee of China Agricultural University. All experiments were conducted in a Biosafety Level 2 (BSL-2) laboratory.

### Prediction of the functional sites in the NDV NP protein

The amino acid sequences of the NDV NP genes were analyzed using three different online programs to identify possible functional sites. PONDR (http://www.pondr.com/) analysis of intrinsically disordered regions (IDRs) was performed to predict the natural structural disorder within the NP protein, while coiled-coil regions of NP were predicted using the COILS program (http://www.ch.embnet.org/software/COILS_form.html)^14,27^. The complete coding sequences of NP from strains belonging to different NDV genotypes were retrieved from the GenBank database (Table S1). The amino acid sequences were analyzed using the ClustalW multiple alignment algorithm in the MegAlign program of the DNASTAR Lasergene package, version 7.1 (DNASTAR Inc. Madison, WI, USA).

### Plasmid construction

The full-length antigenomic cDNA of NDV strain SG10 (rNDV-WT), three helper plasmids encoding the NP, P, and L proteins (pCI-NP, pCI-P, and pCI-L), and the NDV mini-genome plasmid pNDV-MG, containing the firefly luciferase (F-Luc) gene driven by the T7 promoter, were constructed previously^25,45^. pCI-NP cDNA was used as template to PCR-amplify cDNA encoding full-length wild-type NP (489 amino acids) with a Myc tag at its C-terminus, which was cloned into PCI-neo, generating PCI-NP (1-489)-Myc. To generate the mutant constructs, cDNA encoding different mutants with a Myc tag at their C-terminus were cloned into PCI-neo based on the PCI-NP (1-489)-Myc cDNA.

To construct a recombinant cDNA clone containing the E402A mutation within the NP gene, the full-length antigenomic cDNA rNDV-WT was used as the backbone. Briefly, the NP was mutated using overlapping PCR, resulting in the amino acid change E402A. The fragment containing the mutated site was used to replace the corresponding fragment in the full-length cDNA using two unique restriction enzymes, *Xba*I and *Sal*I. The mutated cDNA clone containing the E402A substitution was designated rNDV-NP_E402A_. To analyze the interaction between NP and P protein, plasmids expressing HA-tagged P protein and Flag-tagged NP or NP_E402A_ proteins were constructed by inserting the P or NP/NP_E402A_ genes into the HA-tagged pRK5 vector or Flag-tagged pRK5 vector between the *Eco*RI and *Xba*I sites. All of the constructs were confirmed by nucleotide sequencing.

### NDV mini-genome system and dual luciferase assay

The mini-genome assay was performed as described previously^26^. Briefly, BSR T7 cells were seeded in 24-well culture plates. At 90% confluence, the cells were co-transfected using Lipofectamine 2000 (Invitrogen) with the NDV mini-genome pNDV-MG plasmid carrying the FLuc reporter gene, its helper plasmids, and plasmids encoding untagged NP (PCI-NP), Myc-tagged NP (PCI-NP-Myc), Myc-tagged NP mutants, PCI-P, and PCI-L, as well as a Renilla luciferase reporter gene (RLuc). At 24 h post-transfection, the cells were washed twice in phosphate-buffered saline (PBS), and then lysed in 100 *µ*l of passive lysis buffer (Promega, Madison, WI, USA). Cells were vigorously mixed for 15 min, and 20 *µ*l of lysate were tested using a dual luciferase assay kit (Promega) according to the manufacturer’s instructions to determine the luciferase activity. The relative luciferase activity was defined as the ratio of the FLuc to RLuc activities. The expression of the PCI-NP-Myc and Myc-tagged NP mutant proteins was detected via western blotting with monoclonal anti-Myc antibody sc-40 (Santa Cruz, Dallas, TX, USA). Three separate experiments were performed, and luciferase expression was measured in triplicate in each experiment.

### Recovery of virus from cDNA

The recombinant virus rNDV-NP_E402A_ was recovered by co-transfection of the NDV chimeric full-length cDNA plasmid, along with helper plasmids PCI-NP_E402A_, PCI-P, and PCI-L, into BSR T7/5 cells, as previously described^46^. At 4 days post-transfection, the cells were frozen and thawed three times, and the supernatant was harvested. The supernatant was then injected into the allantoic cavities of 9-day-old SPF embryonated chicken eggs to recover the recombinant NDV. Recovery of the virus was confirmed using a hemagglutination (HA) assay, and the total RNA from NDV-positive allantoic fluid was extracted using TRIzol reagent (Invitrogen) according to the manufacturer’s instructions. The rNDV-NP_E402A_ recombinant was sequenced, and no adventitious mutations were detected.

### Mean death time and intracerebral pathogenicity index of the recombinant viruses

Viral titers of rNDV-WT and rNDV-NP_E402A_ were determined using the Reed and Muench end-point method, and expressed as the 50% embryo infectious dose (EID_50_)/ml and 50% tissue culture infective dose (TCID_50_)/ml^47^. The pathogenicity of the recombinant viruses was determined using the standard pathogenicity tests for NDV: the mean death time (MDT) test in 9-day-old embryonated SPF chicken eggs, and the intracerebral pathogenicity index (ICPI) test in 1-day-old SPF chicks^48^.

### Plaque formation

BSR T7 cells were infected with viruses at a multiplicity of infection (MOI) of 0.01 in six-well plates. After 1 h of adsorption, the inoculum was removed and replaced with an overlay medium containing 2% FBS and 1% agar. Following incubation at 37 °C in a 5% CO_2_ incubator for 36 h, overlay medium supplemented with 0.1% neutral red was added. Plaques were observed after a further 48 h of incubation^49^. Plaque sizes were measured using the GNU Image Manipulation Program, version 2.8 (https://www.gimp.org/)^50^.

### Pathogenicity assessment in 4-week-old chickens

The pathogenicity of rNDV-WT and rNDV-NP_E402A_ was then determined in chickens. Four-week-old SPF white Leghorn chickens were assigned randomly into three groups of 16 birds each (6 for sampling and 10 for clinical observation). Chickens were inoculated via the eye drop/intranasal (ED/IN) route with one of the two recombinant viruses at a dose of 10^5^ EID_50_/100 *μ*l per bird, or with 0.9% NaCl as the negative control. The birds were monitored for clinical signs daily for 14 days post-infection (dpi). Two birds were euthanized daily from 2–5 dpi for gross lesion observation, and samples of the trachea, lung, brain, caecum, spleen, and duodenum were collected and then separated into two parts. One part was used for virus titration in DF-1 cells at 2–4 dpi. These tissue samples were homogenized in phosphate-buffered saline containing with a final concentration of 10,000 units of penicillin G and streptomycin, and the cleared tissue homogenates were serially diluted 10-fold and used to inoculate DF-1 cells. The virus titers were determined as the TCID_50_ per gram using the endpoint method of Reed and Muench^47^. The second part of each tissue sample was fixed in 10% buffered formalin for 48 h for use in histopathology. All of the fixed tissue samples were routinely sectioned and stained with hematoxylin and eosin (H&E), and then examined for lesions using light microscopy (CX31, Olympus, Tokyo, Japan).

### Pathogenicity assessment in 1-day-old chickens

To further compare the pathogenicity of the recombinant viruses, 70 1-day-old SPF chickens were assigned randomly into seven groups of 10 birds each. Each group was separately inoculated via the intracerebral route with rNDV-WT or rNDV-NP_E402A_ at doses of 10^1^ EID_50_/50 *μ*l, 10^2^ EID_50_/50 *μ*l, or 10^3^ EID_50_/50 *μ*l per bird, or with 0.9% NaCl as the negative control. The birds were monitored for clinical symptoms and mortality once every 12 h for 8 days. At each observation, the birds were scored as follows: 0 = normal, 1 = sick, and 2 = deceased. As per the ICPI assay, results were presented as the mean of the score per bird per observation over the eight-day period.

### Western blotting and immunoprecipitation

To determine the role of the glutamic acid at amino acid position 402 in the interaction between NP and P proteins, 293T cells were co-transfected with 2.5 *μ*g of DNA from plasmids expressing HA-tagged P protein, and 2.5 *μ*g of DNA from plasmids expressing Flag-tagged NP or NP_E402A_. At 48 h post-transfection, cells were harvested and analyzed by immunoprecipitation assay. Briefly, the cell lysates were first prepared using lysis buffer (50 mM Tris-Cl at pH 8.0, 150 mM NaCl, 1.0% Triton X-100, 10% glycerol, 20 mM NaF, 1 mM DTT, and 1 × complete protease mixture). Protein samples were resolved by sodium dodecyl sulfate polyacrylamide gel electrophoresis (SDS-PAGE), and then transferred onto a polyvinylidene fluoride membrane (Millipore, Billeriea, MA, USA). The membrane was blocked with 5% skim milk in 0.1% Tween-20/PBS buffer, probed with anti-HA (Santa Cruz) or anti-Flag antibodies (Sigma-Aldrich) at dilution 1:2000, and then incubated with a dilution of 1:10000 of goat anti-mouse or anti-rabbit IgG-HRP (Santa Cruz). Detection was performed using an ECL detection kit (CwBiotech, Beijing, China). The amounts of Flag-tagged proteins were adjusted to similar levels by varying the amounts of the transfected plasmids. The cell extracts were prepared using lysis buffer as described above. Immunoprecipitation assays were then performed with anti-Flag M2 beads (Sigma-Aldrich, St. Louis, MO, USA) for 12 h at 4 °C. Beads were washed three times in lysis buffer and then eluted with sample buffer. Precipitated complexes were detected by Western blotting with anti-HA (Santa Cruz) and anti-Flag antibodies (Sigma-Aldrich).

### Viral replication in DF-1 cells

The growth dynamics of rNDV-WT and rNDV-NP_E402A_ were determined in DF-1 cells under multiple-cycle growth conditions. DF-1 cells in duplicate wells of six-well culture plates were infected with viruses at a MOI of 0.01. After 1 h of adsorption, the cells were washed twice with PBS and then incubated with DMEM containing 2% FBS at 37 °C in a 5% CO_2_ incubator. The culture supernatants were collected and replaced with an equal volume of fresh medium at 12-h intervals until 72 hpi. Virus titers in the collected supernatants were titrated via the limiting dilution method in DF-1 cells, and were expressed as TCID_50_ using the endpoint method^47^. All experiments were performed in triplicate.

### Quantification of viral RNA synthesis by quantitative RT-PCR (qRT-PCR)

DF-1 cells were collected from virus infection assays at the indicated time points (12, 24, and 36 hpi), and total RNA was extracted using 1 ml of TRIzol reagent (Invitrogen) according to the manufacturer’s instructions. The resulting RNA samples (1 *μ*g per sample) were reverse-transcribed using a previously reported method^41^. Briefly, we used the reverse transcription primer PRT-G, which is specific for negative-sense viral RNA (5′-ACG ATA AAA GGC GA AGA AGC A-3′, nucleotide positions 24–44 in the SG10 genome). Reverse transcripts were stored at −70 °C until use in qRT-PCR assays. Primers qNP-F (5′-TTA CAA CTT GGT CGG GGA TG-3′) and qNP-R (5′-CGA TAT AAA CGC ATGA GCT G-3′) were used to quantify the viral NP gene, and primers qP-F (5′-TGG AAG CAA CCA GGG AAG AC-3′) and qP-R (5′-GGC AGG TAG CTG GAC ACG AT-3′) were used to quantify the viral P protein gene. qRT-PCR assays were performed using SYBR Premix Ex Taq (TaKaRa Biotechnology, Shiga, Japan) according to the manufacturer’s instructions. Assays were performed using a LightCycler 96 (Roche, Basel, Switzerland) thermal cycler with the following parameters: 94 °C for 30 s, followed by 40 cycles of 94 °C for 5 s, 60 °C for 10 s, and 60 °C for 15 s. Gene expression was normalized relative to that of the housekeeping gene GAPDH. For data analysis, LightCycler 96 qRT-PCR software, version 1.1.0.1320 (Roche), was used.

### Statistical analyses

All statistical analyses were performed using an unpaired *t*-test in GraphPad Prism Software Version 6.0 (GraphPad Software Inc., San Diego, CA, USA). Statistical differences between experimental groups were determined using the analysis of variance method. Statistical significance was set at *P* < 0.05 (*), *P* < 0.01 (**), and *P* < 0.001 (***).
